# Practical brain MRI guidelines for anti-Aβ antibody treatment in early symptomatic Alzheimer’s disease

**DOI:** 10.1007/s11604-025-01773-x

**Published:** 2025-04-23

**Authors:** Shingo Kakeda, Yukio Miki, Kohsuke Kudo, Harushi Mori, Aya M. Tokumaru, Osamu Abe, Shigeki Aoki, Working Group for Brain MRI Guidelines for Anti-Aβ Antibody Treatment from The Japan Radiological Society, The Japanese Society for Neuroradiology, and The Japanese Society for Magnetic Resonance in Medicine

**Affiliations:** 1 Department of Radiology, Hirosaki University Graduate School of Medicine, Hirosaki, Aomori 036-8562 Japan; 2 Department of Diagnostic and Interventional Radiology, Graduate School of Medicine, Osaka Metropolitan University, Osaka, Japan; 3 Department of Diagnostic Imaging, Faculty of Medicine and Graduate School of Medicine, Hokkaido University, Sapporo, Japan; 4 Department of Radiology, School of Medicine, Jichi Medical University, Tochigi, Japan; 5 Department of Diagnostic Radiology, Tokyo Metropolitan Geriatric Hospital, Tokyo, Japan; 6 Department of Radiology, Graduate School of Medicine, The University of Tokyo, Tokyo, Japan; 7 Department of Neurophysiology, Juntendo University School of Medicine, Tokyo, Japan

**Keywords:** Guidelines, Brain, Magnetic resonance imaging, Anti-amyloid beta antibody treatment, Alzheimer's disease, Amyloid-related imaging abnormalities

## Abstract

**Purpose:**

These guidelines aim to support magnetic resonance imaging (MRI) diagnosis in patients receiving anti-amyloid β (Aβ) antibody treatment without restricting treatment eligibility.

**Materials and methods:**

These guidelines were collaboratively established by Japan Radiological Society, The Japanese Society of Neuroradiology, and Japanese Society for Magnetic Resonance in Medicine by reviewing existing literature and the results of clinical trials.

**Results:**

Facility standards should comply with the “Optimal Use Promotion Guidelines” of Japan, and physicians should possess comprehensive knowledge of amyloid-related imaging abnormalities (ARIA) and expertise in brain MRI interpretation. The acquisition of knowledge regarding amyloid-related imaging abnormalities, brain MRI, anti-Aβ antibody introduction, and post-treatment diagnosis are also recommended.

**Conclusion:**

These guidelines facilitate the accurate diagnosis and effective management of ARIA; ensure the safe administration of anti-Aβ drugs; and provide a framework for MRI facilities, includes staffing requirements and the use of MRI management systems.

**Supplementary Information:**

The online version contains supplementary material available at 10.1007/s11604-025-01773-x.

## Introduction

For the treatment of early Alzheimer’s disease (AD), the Food and Drug Administration approved anti-amyloid β (Aβ) monoclonal antibodies: lecanemab (LeqembiTM) on July 6, 2023, and donanemab (Kisunla) on July 2, 2024. In Japan, lecanemab was approved by the Japanese regulatory authorities on September 25, 2023, and was covered by the Japanese health insurance on December 20 [1]. More recently, in Japan, medication coverage for donanemab became available on November 24, 2024. Randomized clinical trials have shown that the use of anti-Aβ antibodies is associated with magnetic resonance imaging (MRI) signal abnormalities treatment-related adverse events called amyloid-related imaging abnormalities (ARIA) [2–11]. Given that the presence of any hemorrhage on initial MRI (anti-Aβ antibody introduction MRI) increases the risk of ARIA [12], the detection of cerebral microbleeds and superficial hemosiderosis on initial MRI is crucial. During drug treatment, ARIA detection and severity assessment are vital for determining whether to continue, suspend, discontinue, or resume therapy. Consequently, brain MRI plays a vital role in patients considered for anti-Aβ antibody treatment. The roles of MRI include the following: (1) assessment of eligibility for therapy, including differential diagnosis of dementia for initial MRI, and (2) detection and diagnosis of ARIA for follow-up (on- or post-treatment) MRI. Diagnostic imaging physicians are required to possess comprehensive knowledge of ARIA and general skills in interpreting brain MRI findings, including cerebrovascular diseases, brain tumors, inflammatory conditions, traumatic brain injuries, metabolic diseases, and malformations. Although Japan has the highest MRI accessibility worldwide, there are relatively few radiologists specializing in neuroradiology who can detect subtle abnormalities [13].

Prompt diagnosis and management of ARIA are essential for the safe administration of anti-Aβ drugs. ARIA is often characterized by subtle abnormalities, emphasizing the need for quality control of MRI. Considering that patients with ARIA may require urgent attention, an institutional framework for MRI facilities is essential. This framework includes staffing requirements, such as diagnostic imaging physicians and radiological technologists, as well as an MRI management system that can maintain appropriate imaging protocols. Although there are reviews and guidelines on the radiological, biological, and clinical characteristics of ARIA [12, 14], a few reviews discuss the differential diagnosis of dementia and ARIA on initial MRI in treated patients. Furthermore, there are no practical guidelines addressing facility requirements, qualifications for diagnostic imaging physicians, and required knowledge. Therefore, Japan Radiological Society (https://www.radiology.jp/english/index.html), The Japanese Society of Neuroradiology (https://neurorad.jp/english-page/), and Japanese Society for Magnetic Resonance in Medicine (https://www.jsmrm.jp/modules/en/index.php?content_id=1) collaboratively present these guidelines for brain MRI examination and MRI-based diagnosis in patients undergoing anti-Aβ antibody treatment.

## Framework for MRI diagnosis

### Institutional requirements

Facility standards should comply with the “Optimal Use Promotion Guidelines,” which are national regulations in Japan aimed at ensuring the appropriate use of medical imaging resources. MRI facilities should strive to maintain management systems equivalent to Added fees for Radiological Managements on Imaging-studies (ARMIs) 3 or 4, with full-time radiologists*. A collaborative system between prescribing physicians and brain MRI diagnosticians is essential, and efforts should be made to conduct examinations using protocols that are standardized across the same MRI equipment whenever possible.

### Diagnostic imaging physician qualifications

Diagnostic imaging physicians should possess expertise in brain MRI interpretation, encompassing not only dementia-related findings but also cerebrovascular lesions, brain tumors, inflammatory diseases, traumatic injuries, metabolic conditions, and congenital anomalies.

Diagnostic imaging physicians should explain the importance and necessity of appropriate protocols to radiological technologists (primarily referring to Magnetic Resonance Technological Specialists**) and optimize ARIA imaging protocols with radiological technologists at each facility. Diagnostic imaging physicians should establish STAT reporting systems*** for urgent MRI interpretation in accordance with guidelines from relevant societies (The Japan Radiological Society and The Japanese College of Radiology) and maintain collaboration with radiological technologists for emergency findings.

To ensure continuity of MRI examinations, consideration should be given to conducting MRI examinations at affiliated facilities when both the quality of examinations and diagnostic imaging are assured, similar to amyloid positron emission tomography (PET) or cerebrospinal fluid (CSF) examinations.

## Knowledge requirements for diagnostic imaging physicians

Variations in MRI equipment among vendors, magnetic field strength, and MRI sequences influence the image findings of ARIA. Therefore, the three MRI-related academic societies (The Japan Radiological Society, The Japanese Society for Neuroradiology, and The Japanese Society for Magnetic Resonance in Medicine) propose acquiring the following knowledge:

### About ARIA [5, 8, 12, 14–16]

- Classification (ARIA-E and ARIA-H)
- Pathophysiology (incidence, clinical presentation, timing, and progression)
- Diagnostic criteria, severity classification, and management strategies.

### Brain MRI

Although protocols are not fully established, Table 1 shows the recommended protocol by the three societies based on phase III clinical trials. Baseline and follow-up MRI should use identical imaging parameters on the same equipment. For brain MRI diagnosis, knowledge of the following imaging sequences is recommended:

- Differences between 3 and 2D imaging (T1 weighted imaging, fluid-attenuated inversion recovery [FLAIR], etc.) [17]
- Basic knowledge of T2* gradient-echo (GRE) sequence and susceptibility-weighted imaging (SWI) sequence [18]
- Knowledge of imaging protocol parameters (e.g., repetition time [TR], echo time [TE], and inversion time [TI]) and their impact on image visualizations
- Impact on image visualization by MRI equipment variations among vendors, and relationship between field strength and image visualizations (1.5 T vs. 3 T equipment)
- Relationship between imaging parameters (TR, TE, and TI) and image visualizations (e.g., FLAIR) [17, 19], relationship between slice thickness and lesion detection [20]
- Knowledge regarding diffusion-weighted imaging (DWI) and apparent diffusion coefficient map [12]
- Relationship between parallel imaging and acquisition time/image quality [21, 22]
- Knowledge of vendor-specific imaging methods, their sequence names, visualization capabilities, and artifacts (particularly for SWI, similar sequences include the Principle of Echo Shifting with a Train of Observation and T2 Star Weighted magnetic resonance Angiography)
- Knowledge of brain volume analysis (voxel-based morphometry [23–26]), advanced imaging techniques (e.g., compressed sensing [27], AI-based noise reduction [28, 29]), and pathophysiological analysis using brain MRI (e.g., diffusion tensor imaging, diffusion tensor image analysis along the perivascular space: Diffusion Tensor Imaging–Analysis Along the Perivascular Space method) [30, 31].

**Table 1:** MRI sequences to be included in the imaging protocol (as recommended by three societies^a^)

MRI sequences	Purpose	Initial (baseline)	Follow-up (asymptomatic)	Follow-up (symptomatic)
3D-T1WI	Brain volume evaluation (atrophy)	〇	△	△
DWI	Differential diagnosis of acute cerebral infarction	◎	◎	◎
T2WI	Detection of various lesions	〇		〇
FLAIR	Diagnosis of ARIA-E	◎	◎	◎
T2* GRE or SWI^b^	Diagnosis of ARIA-H	◎	◎	◎
T2WI (Cor)	Evaluation of hippocampal atrophy	〇		
MR angiography	Evaluation of vascular lesions (stenosis and aneurysms)	〇		〇
Gd-T1WI^c^	Detection of enhanced brain lesions (metastases, meningitis, etc.)			△

*DWI* diffusion-weighted imaging, *FLAIR* fluid-attenuated inversion recovery, *GRE* gradient-echo, *SWI* susceptibility-weighted imaging

◎ = essential MRI sequence, 〇 = recommended MRI sequence, △ = optional MRI sequence, blank field: omissible MRI sequence

^a^Japan Radiological Society, The Japanese Society for Neuroradiology, and Japanese Society for Magnetic Resonance in Medicine

^b^For ARIA-H evaluation, SWI can also be used instead of T2* GRE. Although SWI has higher sensitivity for detecting cerebral microbleeds compared to T2* GRE, SWI-capable equipment may be limited

^c^Additional examinations with gadolinium contrast may be performed when further lesion differentiation is needed

### Initial MRI (anti-Aβ antibody introduction) diagnosis (Table 2)

**Table 2:** Diseases and conditions included in the initial (drug introduction) MRI diagnosis

Diseases and conditions suggesting non-AD dementia with clinical significance	Diseases and conditions impacting treatment efficacy with significant role of diagnostic imaging
Degenerative dementia	Amyloid angiopathy-related inflammation^a^
Alzheimer’s disease	Vascular lesions (cerebral aneurysm, arteriovenous fistula)
Lewy body disease, dementia with Parkinson's	Idiopathic normal pressure hydrocephalus (adult chronic hydrocephalus Hakim's disease)
Argyrophilic grain disease	Vascular malformations: cavernous hemangioma, arteriovenous malformation, venous malformation
Frontotemporal lobar degeneration (FTLD)	Brain tumors^b^
Progressive supranuclear palsy (PSP)	PML
Corticobasal degeneration (CBD)	Neurosyphilis
Senile dementia of the neurofibrillary tangle (SD-NFT)	Epilepsy
Globular glial tauopathy (GGT)	Alcohol-related
Chronic traumatic encephalopathy	Post-seizure encephalopathy
	AIDS-related encephalopathy
Vascular dementia	Neuroinfection
Large territory infarction, hemorrhage	Vasculitis
Binswanger’s disease	Demyelinating diseases
Strategic infarct dementia	Toxicity: carbon monoxide poisoning, drugs, metals
Cortical microinfarcts	Prion disease
Cerebral amyloid angiopathy	Neuronal intranuclear inclusion disease
Hypoperfusion	ALSP/HDLS-CSF1R
Chronic subdural hematoma	White matter diseases (various pathologies)
	Traumatic brain injury (TBI)
	Brain contusion, diffuse axonal injury, chronic subdural hematoma, chronic traumatic encephalopathy (CTE)
	Nutritional/metabolic disorders: vitamin B1 deficiency, vitamin B12 deficiency, folate deficiency, etc
	Organ failure and related diseases: hepatic encephalopathy, renal failure

*PML* progressive multifocal leukoencephalopathy, *AIDS* acquired immune deficiency syndrome, *ALSP* adult-onset leukoencephalopathy with axonal spheroids and pigmented glia, *HDLS-CSF1R* hereditary diffuse leukoencephalopathy with spheroid due to *CSF1R* mutations

^a^Considered to have similar pathology to ARIA

^b^Meningiomas or arachnoid cysts diagnosed as lesions with maximum diameters < 1 cm are not excluded for drug administration

Assessment should be made regarding contraindications for anti-Aβ antibody introduction (check Sheet A in Supplementary material). In addition to diagnosing ARIA (spontaneous ARIA), diagnostic imaging physicians must perform standard brain MRI diagnosis. Therefore, knowledge of MRI findings [32, 33] for AD and non-AD conditions affecting cognitive function is required. The cognitive dysfunction-presenting non-AD degenerative diseases include Lewy body dementia [34, 35], frontotemporal lobar degeneration [36], argyrophilic grain disease, corticobasal degeneration [37], cerebral infarction/hemorrhage [38, 39], subdural hematoma, encephalitis/encephalopathy, traumatic brain injury (TBI) (brain contusion, diffuse axonal injury [DAI]) [40, 41], idiopathic normal pressure hydrocephalus (adult chronic hydrocephalus Hakim’s disease) [42], brain tumors (hemorrhagic metastases), vascular malformations/hemangiomas (cavernous hemangioma), vascular lesions (cerebral aneurysms, arteriovenous malformation/fistulas), inflammatory diseases (infection, autoimmune encephalopathy), cerebral amyloid angiopathy (CAA) [43, 44], neuronal intranuclear inclusion disease [45], and others.

### Follow-up MRI (on- or post-treatment) diagnosis

Refer to Check Sheet B (Supplementary material) created by the three societies for ARIA-E and ARIA-H detection and severity assessment. Before MRI interpretation, diagnostic imaging physicians should confirm whether the imaging equipment and conditions match the initial MRI, including TR, TE, TI, and spatial resolution parameters. MRI diagnosis includes regular follow-up, follow-up for patients with ARIA, and emergency MRI for symptomatic cases. Regular follow-up includes initial MRI diagnosis plus ARIA-E and ARIA-H detection and severity assessment (number, extent, and size of lesions, if detected). For patients with ARIA, follow-up includes the evaluation of ARIA changes. Emergency MRI requires sequence selection (potentially including contrast) and differential diagnosis based on symptoms (Tables 2, 3, 4).

**Table 3:** Differential diagnoses of ARIA-E, pathologies/diseases, and MRI findings

ARIA-E (brain parenchymal edema) and similar findings	ARIA-E (sulcal effusion) and similar findings
MR sequence related/anatomical causes	MR sequence related/anatomical causes
Signal changes due to imaging parameter settings	Water signal suppression failure
	Susceptibility artifacts (mostly iatrogenic):
Pathologies/diseases	Metal (dental work, clips, coils), oxygen administration, recent Gd contrast administration, increased blood pool ratio
Acute cerebral infarction (arterial, venous)	
Brain tumors (metastatic, primary), bloomy rind sign	Motion Artifacts
Traumatic brain injury (TBI)	CSF inflow, vascular pulsation, body movement
Brain edema from extra-axial masses	
Infectious encephalitis, metabolic encephalopathy	Pathologies/Diseases
Autoimmune encephalitis/encephalopathy	Accumulation
Epilepsy, familial hemiplegic migraine	Subarachnoid hemorrhage, fat components, amyloid
Progressive multifocal leukoencephalopathy (PML)	Infiltration:
Posterior reversible encephalopathy syndrome (PRES)	Meningeal carcinomatosis, melanomatosis, sarcoidosis
	Flow
	Intravascular signals (embolism, ivy sign, hyperperfusion)
	Meningitis and its sequelae

**Table 4:** Differential diagnoses of ARIA-H, pathologies/diseases, and MRI findings

ARIA-H (cerebral microbleeds, hemorrhage)	ARIA-H (superficial hemosiderosis)
MR sequence related/anatomical causes	MR sequence related/anatomical causes
Normal vessels (vascular signal void)	Skull base susceptibility artifacts
Physiological calcification	
	Pathologies/Diseases
Pathologies/Diseases	Subarachnoid hemorrhage
Conditions with calcification	Trauma
Cavernous hemangioma/vascular malformation	Cerebral aneurysm, arteriovenous malformation
Hypertensive microbleeds and cerebral amyloid angiopathy	Cerebral venous thrombosis
Embolic microbleeds: fat, air, IE, sepsis, HbS disease, ITP	RCVS
Traumatic brain injury (TBI):	Remote cerebellar hemorrhage
Diffuse axonal injury (DAI), cerebral small vessel injury	Vasculitis
Brain contusion	Spinal duropathy
Brain tumors	Pseudo laminar cortical necrosis
Hemorrhagic metastases: melanoma, renal, thyroid, lung, myxoma	Post-infarct cortical hemorrhagic transformation
Vasculitis	Subdural hematoma-related
PCNSV, secondary: infection, tumor, drug-related	
Post-radiation	
Hereditary	
CADASIL/CARASIL, *COL4A1*-related diseases, Fabry disease	

*HbS* sickle cell disease, *IE* infective endocarditis, *ITP* idiopathic thrombocytopenic purpura, *CADASIL* cerebral autosomal dominant arteriopathy with subcortical infarcts and leukoencephalopathy, *CARASIL* cerebral autosomal recessive arteriopathy with subcortical infarcts and leukoencephalopathy, *PCNSV* primary central nervous system vasculitis, *RCVS*, reversible cerebral vasoconstriction syndrome

#### Knowledge required for ARIA-E assessment (Table 3)

- FLAIR sequence–related/anatomical causes [46]: false positives due to water signal suppression failure, artifacts from air, bone (skull base), foreign objects (metals etc.), oxygen administration, increased blood pool ratio, recent Gd contrast administration, and motion artifacts (CSF inflow, vascular pulsation, body movement)
- Differentiation between vasogenic and cytotoxic edema on DWI [47]
- ARIA-E (edema) differential diagnoses [11, 48, 49]: acute cerebral infarction, posterior reversible encephalopathy syndrome, progressive multifocal leukoencephalopathy, encephalitis/encephalopathy (infectious, non-infectious, metabolic, autoimmune), brain tumors (primary brain tumors, edema due to extra-axial masses, metastatic brain tumors), TBI, and epilepsy-related brain changes
- ARIA-E (effusion) differential diagnoses [11, 48, 49]: subarachnoid hemorrhage, meningitis and its sequelae, high signal in sulci associated with cerebral artery occlusion (ivy sign).

#### Knowledge required for ARIA-H assessment (Table 4)

- T2*GRE and SWI sequence related/Anatomical causes [50]: normal vessels (vascular signal void), physiological calcification, susceptibility artifacts (e.g., skull base)
- Diagnosis of microbleeds, superficial hemosiderosis, and cerebral hemorrhage. In the diagnosis of microbleeds, although the cause (hypertensive or CAA) is clinically important, the anatomical location (etiology of microbleeds) is not considered in the severity classification of ARIA-H.
- ARIA-H (intracerebral hemorrhage) differential diagnoses [51]: cavernous hemangioma/vascular malformation, TBI (brain contusion, DAI)
- ARIA-H (superficial hemosiderosis) differential diagnoses [52]: subarachnoid hemorrhage, post-traumatic changes, and subdural hematoma and its history [53].

## Final notes

These guidelines aim to support MRI diagnosis in patients receiving anti-Aβ antibody treatment without restricting treatment eligibility. Although current guidelines address the latest approved drugs, the basic principles will apply to new medications, with updates planned as new information becomes available. Therefore, regular updates are necessary for aspects outside these guidelines, including amyloid PET examination and ARIA management.

## Annotations

* ARMIs: A supplementary payment system in the Japanese medical insurance system to ensure quality of diagnostic imaging. The system has four tiers (1–4) based on facility implementation and management systems. One of the requirements for ARMIs 3 and 4 is the presence of three or more and six or more full-time diagnostic imaging physicians, respectively.

** Magnetic Resonance Technological Specialist: A technologist in Japan certified by the Japan Authorize Organization for Magnetic Resonance Technological Specialist who works with magnetic resonance examinations, with the purpose of ensuring international equivalency of magnetic resonance examination techniques, providing standardized optimal imaging information that accommodates the latest medical technologies, and guaranteeing safety.

*** STAT Image Reporting: Refers to the act of radiological technologists providing findings of STAT images to physicians according to the “Guidelines for Reporting STAT Image Findings of High-Emergency Diseases Affecting Life Prognosis” by the Japan Radiological Society and the Japanese Society of Radiological Technology. STAT images refer to “images showing findings of high-emergency diseases affecting life prognosis,” derived from the Latin word “statim,” which directly translates to “immediately.”

## Supplementary Information

Below is the link to the electronic supplementary material.

Supplementary file1 (DOCX 26 KB)
