# Supplementary material for: Practical brain MRI guidelines for anti-Aβ antibody treatment in early symptomatic Alzheimer’s disease
Source: Jpn J Radiol. 2025 Apr 23;43(8):1231–8. doi: 10.1007/s11604-025-01773-x (PMC12287243; doi:10.1007/s11604-025-01773-x)
Supplement: Supplementary file 1 — Supplementary file1 (DOCX 26 KB) [file 11604_2025_1773_MOESM1_ESM.docx]

**Check Sheet A (Pre-Treatment Screening)**

**1. Contraindications for Drug Administration**

- Cerebral microbleeds (maximum diameter < 10 mm) at ≥5 locations: ( )
- Cortical superficial siderosis: ( )
- Cerebral hemorrhage (maximum diameter ≥ 10 mm): ( )
- Vasogenic edema: ( )

**2. Considerations for Drug Administration**

- Clinically significant lesions suggestive of dementia other than AD: ( )
- Brain contusion, encephalomalacia: ( )
- Aneurysm, vascular malformation: ( )
- Infectious lesions: ( )
- Severe small vessel disease, severe white matter lesions: ( )
- Space-occupying lesions: ( )

**Check Sheet B (Monitoring MRI On-Treatment)**

**Comparison to Previous MR Images (FLAIR, T2*WI, SWI)**

- Same MRI equipment (vendors etc.): (Yes or No)
- Differences in magnetic field strength of the MRI system (3T or 1.5T): ( )
- Differences in slice thickness (FLAIR, T2*WI, SWI): ( )
- Differences in imaging parameters (e.g., TE in T2*WI, SWI): ( )

**ARIA Assessment**

**ARIA-E**

1. New incident ARIA-E:
   1. Mild: ( ); Moderate: ( ); Severe: ( )
2. Follow-up for previously identified ARIA-E:
   1. ( ) Resolution
   2. ( ) Persistence (Mild, Moderate, Severe)

**ARIA-H**

1. New incident ARIA-H:
   1. Mild: ( ) (cerebral microbleeds, cortical superficial siderosis)
   2. Moderate: ( ) (cerebral microbleeds, cortical superficial siderosis)
   3. Severe: ( ) (cerebral microbleeds, cortical superficial siderosis, cerebral hemorrhage)
2. Follow-up for previously identified ARIA-H:
   1. ( ) Stabilization (cerebral microbleeds, cortical superficial siderosis, cerebral hemorrhage)
   2. ( ) Progression (cerebral microbleeds, cortical superficial siderosis, cerebral hemorrhage)

*Note: Stabilization = No progression of ARIA-H

**Appendix**

**Severity Classification for ARIA-E**

- **Mild**: Size (<5 cm), Location (a single site within sulcus or cortex/subcortical white matter)
- **Moderate**: Size (5–10 cm), Location (one or multiple brain locations)
- **Severe**: Size (>10 cm)

Note: Anatomical location is not considered; if a lesion spans multiple contiguous regions, it is treated as one lesion. However, if a lesion crosses both hemispheres, it is divided at the midline for separate evaluation.

**Severity Classification for ARIA-H**

- **Mild**:
  1. New incident cerebral microbleeds (1–4)
  2. Cortical superficial siderosis at 1 location
- **Moderate**:
  1. New incident cerebral microbleeds (5–9)
  2. Cortical superficial siderosis at two locations
- **Severe**:
  1. New incident cerebral microbleeds (≥10)
  2. Cortical superficial siderosis at ≥3 locations

Note: If severity differs between cerebral microbleeds and cortical superficial siderosis, the higher severity classification is applied.
